# Effects of the Commercial Flame Retardant Mixture DE-71 on Cytokine Production by Human Immune Cells

**DOI:** 10.1371/journal.pone.0154621

**Published:** 2016-04-29

**Authors:** Thit Mynster Kronborg, Juliana Frohnert Hansen, Claus Henrik Nielsen, Louise Ramhøj, Marie Frederiksen, Katrin Vorkamp, Ulla Feldt-Rasmussen

**Affiliations:** 1 Department of Medical Endocrinology, PE 2132, Rigshospitalet, University of Copenhagen, Copenhagen, Denmark; 2 Institute for Inflammation Research, section 7521, Rigshospitalet, University of Copenhagen, Copenhagen, Denmark; 3 National Food Institute, Division of Diet, Disease Prevention and Toxicology, Technical University of Denmark, Soeborg, Denmark; 4 Department of Energy, Environment and Indoor Climate, Danish Building Research Institute, Aalborg University, A.C. Meyers Vænge 15, 2450 København SV, Denmark; 5 Department of Environmental Science, Aarhus University, Frederiksborgvej 399, 4000 Roskilde, Denmark; Emory University, UNITED STATES

## Abstract

**Introduction:**

Although production of polybrominated diphenyl ethers (PBDEs) is now banned, release from existing products will continue for many years. The PBDEs are assumed to be neurotoxic and toxic to endocrine organs at low concentrations. Their effect on the immune system has not been investigated thoroughly. We aimed to investigate the influence of DE-71 on cytokine production by peripheral blood mononuclear cells (PBMCs) stimulated with *Escherichia Coli* lipopolysaccharide (LPS) or phytohaemagglutinin-L (PHA-L).

**Material and Methods:**

PBMCs isolated from healthy donors were pre-incubated with DE-71 at various concentrations and subsequently incubated with the monocyte stimulator LPS, or the T-cell activator PHA-L. Interferon (IFN)-γ, interleukin (IL)-1β, IL-2, IL-4, IL-6, IL-8, IL-10, tumor necrosis factor (TNF)-α, IL-17A, and IL-17F were quantified in the supernatants by Luminex kits.

**Results:**

At non-cytotoxic concentrations (0.01–10 μg/mL), DE-71 significantly enhanced secretion of IL-1β, IL-6, CXCL8, IL-10, and TNF-α (p<0.001–0.019; n = 6) from LPS-stimulated PBMCs. IFN-γ, TNF-α, IL-17A, and IL-17F (p = <0.001–0.043; n = 6) secretion were enhanced from PHA-L-stimulated PBMCs as well. Secretion of IL-1β, IL-2, IL-10, IL-8 and IL-6 was not significantly affected by DE-71.

**Conclusions:**

We demonstrate an enhancing effect of DE-71 on cytokine production by normal human PBMCs stimulated with LPS or PHA-L *ex vivo*.

## Introduction

Polybrominated diphenyl ethers (PBDEs) are used in flame retardant mixtures to reduce flammability in electronic equipment, upholstery, textiles, plastics, and building materials [1]. Commercial production of the lower brominated PentaBDE and OctaBDE mixtures was banned in the European Union (EU) in 2004, and phased out in the USA in 2005 [1–4] due to increasing concerns about adverse physiological effects and biological persistence of the compounds [5–7]. The Penta- and OctaBDE mixtures have been on the Stockholm convention list since 2009, giving them status as global persistent organic pollutants. However, because of a long life-time of some products, PBDEs will continue to leak from existing products for many years [1]. Ongoing human exposure in combination with year-long PBDE elimination half-lives [8] will result in substantial PBDE accumulation in humans in the future. One of the commercial mixtures of PBDEs was the PentaBDE mixture DE-71. DE-71 consists of a specific combination of congeners similar to the combination found in dust and indoor air, two potential exposure pathways. This combination of congeners can still be recognised in human samples, such as blood, placenta, amniotic fluid, and breast milk [9, 10]. The PBDEs are assumed to be neurotoxic, and toxic to reproductive and other endocrine organs at low concentrations [1, 11, 12]. Besides, they have been suggested to have immune modulating effects [13–16].

One of many measurable immunologic end points is cytokine secretion [15, 17–20]. Tumor necrosis factor-α (TNF-α), interleukin-1β (IL-1β), and IL-6 characterise pro-inflammatory, innate immune reponses, while interferon-γ (IFN-γ), IL-2, IL-13 and IL-17 characterise T-cell responses, and thereby adaptive immune responses.

The aim of the present study was to investigate the influence of DE-71 on cultures of human peripheral blood mononuclear cells (PBMCs) stimulated with *Escherichia (E*.*) coli* lipopolysaccharide (LPS), an activator of monocytes/macrophages, or the T-cell activator phytohaemagglutinin-L (PHA-L). Unlike most other studies on cellular toxicity, we measured PBDE levels in selected samples of the stock solutions, culture media as well as in supernatant samples and cell remnants after DE-71 exposure.

## Material and Methods

### Subjects

Blood samples were drawn from a total of six healthy, anonymous, female and male volunteers at Rigshospitalet. Fresh blood was collected in sodium-heparin tubes (Vacutainer, Becton Dickinson, Franklin Lakes, NJ) 20 to 30 minutes prior to initiation of the experiment.

### Cell Cultures

PBMCs were isolated by density centrifugation (Ficoll-Hypaque, Almeco, Esbjerg, Denmark and Lymphoprep, Axis-Shield, Oslo, Norway), washed thrice in phosphate buffered saline (PBS without calcium and magnesium from Gibco, Invitrogen, Thermo Fischer Scientific, Waltham, MA), and re-suspended in Hams F12 growth media (Panum Institute, Copenhagen, Denmark), supplemented with 5% foetal bovine serum (Biological Industries, Beit HaEmek, Israel), 0.029% L-glutamin (Panum Institute, Copenhagen University, Denmark), non-essential amino acids and Penicillin/Streptomycin (Gibco, Invitrogen, Thermo Fischer Scientific, Waltham, MA). All experiments consisted of three 24 well plates with final concentrations of 10^6^ cells per well (NUNC, Roskilde, Denmark).

### DE-71 and Stimulation

DE-71, a gift kindly provided by Marta Axelstad, National Food Institute, Technical University of Denmark and Dr. Kevin Crofton of the U.S. Environmental Protection Agency, was dissolved in dimethyl sulfoxid (DMSO) (D2438 Sigma Aldrich, St. Louis, MO) and diluted in cell culture medium to final concentrations of 0.01, 0.1, 1, 5, and 10 μg/mL, respectively. Three standards in DMSO and two standards in cell culture medium were analysed to determine PBDE concentrations, as described below. The DMSO concentration in each well was 1‰. Cell cultures with DE-71 were pre-incubated for one hour at 37°C and 5% CO_2_. Subsequently, LPS from *E*. *coli* (100 pg/mL) or PHA-L (5 μl/mL, both with CAS 9008-97-3, from Sigma-Aldrich, St. Louis, MO) was added. Unstimulated PBMCs, i.e. cell cultures exposed to DE-71 but without LPS or PHA-L stimulation, were also investigated. Cultures were further incubated for 20 to 22 hours, centrifuged for 10 min. at 460 x G at 4°C, and supernatants were harvested and stored at -20°C. All measurements of DE-71 were performed in duplicate, except for two samples where only single determinations were done (one sample of 0.1 μg/mL DE-71; one DMSO control). All experiments included a culture medium-control and a culture medium-control with 1 ‰ DMSO added. The 1 ‰ DMSO control was used for statistical analysis.

### Cytokine Assessment

The content of IFN-γ, IL-1β, IL-2, IL-4, IL-6, IL-8, IL-10, TNF-α, IL-17A, and IL-17F in PBMC culture supernatants after 18 hours of incubation was measured by luminex kits (Biorad Laboratories Inc., Hercules, CA) according to the manufacturer’s protocol. A LUMINEX 100 system (Luminex Cooperation, Austin, TX) was used for the analysis. The intra- and inter-assay variations (% CV) were 2–3% and 3–8% for the chemokine kit, and 4.3% and 5.8% for the Th17 cytokine kit, respectively, as stated by the manufacturer. The calibration ranges in the assays were specific for each cytokine (total range: 0.4–38,787 pg/mL).

### Cytotoxicity Assessment

DE-71 induced cytotoxicity was analysed by assessing the content of lactate dehydrogenase (LDH) in cell culture supernatants as described [21]. For this purpose, a homogenous membrane integrity assay, CytoTox-ONE^™^ (Promega, Fitchburg, WI), was applied according to the manufacturer’s protocol with the modification that LDH was not measured directly in cell cultures but in harvested supernatants. The CytoTox-ONE^™^ passes the following quality control, according to the manufacturer: average background < 20% of the average relative flouroscense unit generated by LDH equivalent to 50,000 cells, LDH activity equivalent to 1,562 cells, fluorescence produced by LDH activities ranging from 0 to 50,000 cells with an R2 ≥ 0.95. Briefly, frozen harvested cell culture supernatants were thawed, mixed by vortex, stimulated with LPS or PHA-L, and exposed to 0.01, 0.1, 1, 5, 10, and 50 μg/mL DE-71 (n = two blood cell cultures set up in triplicates). Harvested cell culture supernatants from the culture medium control with or without DMSO served as negative control. Fifty μl of each culture medium were transferred to a 96-well microwell plate (Th. Geyer, Renningen, Germany), followed by addition of 50 μl CytoTox-One reagent (Promega, Fitchburg, WI) to all wells. The microwell plate was mixed gently on a shaker and incubated at room temperature for 10 to 15 minutes. Hereafter, 25 μl stop solution was added, the plate was shortly shaken, and results were read on a fluorometer (Victor2, PerkinElmer, Waltham, MA). The LDH contents were proportional to the generated fluorescence (given in relative flourescense units), which was used to evaluate the toxicity of DE-71. The DMSO-concentration was 1 ‰ in the negative culture medium control and in cell cultures, to which 50, 10 or 5 μg/mL DE-71 had been added. In samples/cell cultures where 1, 0.1 or 0.01 μg/mL DE-71 had been added, the DMSO concentrations were 0.2‰, 0.02‰ and 0.002‰, respectively.

### Endotoxin Test

All reagents were tested for endotoxins by the Limulus Amebocyte Lysate QCL-1000 assay (Lonza, Basel, Schwitzerland).

### PBDE Analysis

Two supernatants and two cell samples (addition of DE-71 at concentrations of 10 μg/mL and 0.01 μg/mL respectively), two controls (1 ‰ DMSO), three stock solutions of DE-71 in DMSO, and two solutions of DE-71 in culture media were analysed for content and composition of BDE-congeners. This was done after the cytokine analysis using once-thawed samples. The analysis followed accredited methods for PBDEs in biota, as described elsewhere [22], and included 11 tri- to heptabrominated congeners (BDE-17, 28, 47, 49, 66, 85, 99, 100, 153, 154 and 183). Between batch analyses of the in house reference material (n = 18), sand eel oil, varied from 2.7% (BDE-47) to 14% (BDE-17), with a mean of 6.6% for all congeners. The detection limits of the instrument ranged between 0.05 and 0.25 pg. Briefly, the samples were spiked with recovery standards, dried with diatomaceous earth (Varian) and Soxhlet extracted with hexane:acetone (4:1). The extracts were cleaned up on a multilayer column consisting of aluminium oxide, silica, and acidified silica. After elution, volume reduction and addition of the internal standard (BDE-71, Cambridge Isotope Laboratories, Tewksbury, MA), the samples were analysed by gas chromatography—mass spectrometry (GC-MS) with electron capture negative ionization. Quantification was based on two calibrations of ten standards each (0.05–25 ng/mL). Three spiked control samples were extracted together with the samples. The DE-71 standards in DMSO, along with four spiked control samples, were evaporated to dryness in silicone vials [22], re-dissolved in iso-octane including the internal standard and analysed without further clean-up.

### Ethics

The study was approved by The Danish committees on health research ethics, Capital region (Protocol number: H-1-2012-110 and additional protocol 44717), which in Denmark/Copenhagen also functions as the institutional review board. According to the committee law by the Danish Committees on Health Research Ethics, neither written nor oral informed consent is needed in studies of anonymous human blood samples, which was the case in this study. Blood samples of this study were drawn from anonymous healthy human volunteers, whose identity were unknown to the investigators, and thus the institutional review board waived the need for written informed consent from the participants.

### Statistical Analysis

**Fig 1:**
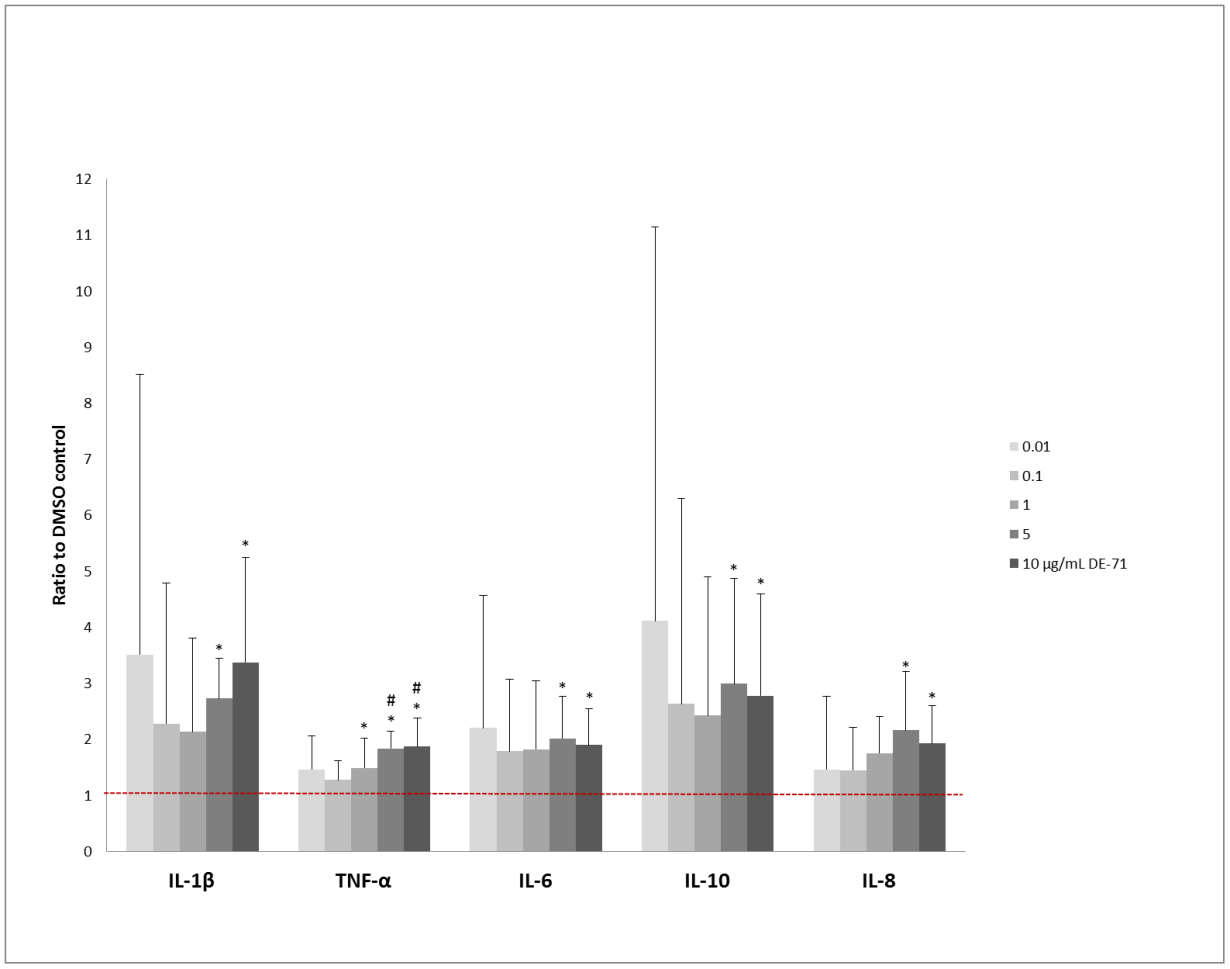
Influence of DE-71 on cytokine responses of PBMCs. Mean + SD of secretion of IL-1β, IL-10, TNF-α, IL-8, and IL-6 from DE-71 exposed and LPS-stimulated PBMCs are shown as ratios to the respective DMSO controls. The red dotted line indicates the level of the DMSO controls (ratio = 1). * = p<0.05 compared to DMSO control, # = p<0.05 compared to 0.1 μg/mL DE-71. PBMC: peripheral mononuclear cells, LPS: lipopolysaccharide, IL-1β: interleukin-1β, IL-10: interleukin-10, TNF-α: tumor necrosis factor-α, IL-8: interleukin-8, IL-6: interleukin-6, DMSO = dimethyl sulfoxide, SD = standard deviation.

**Fig 2:**
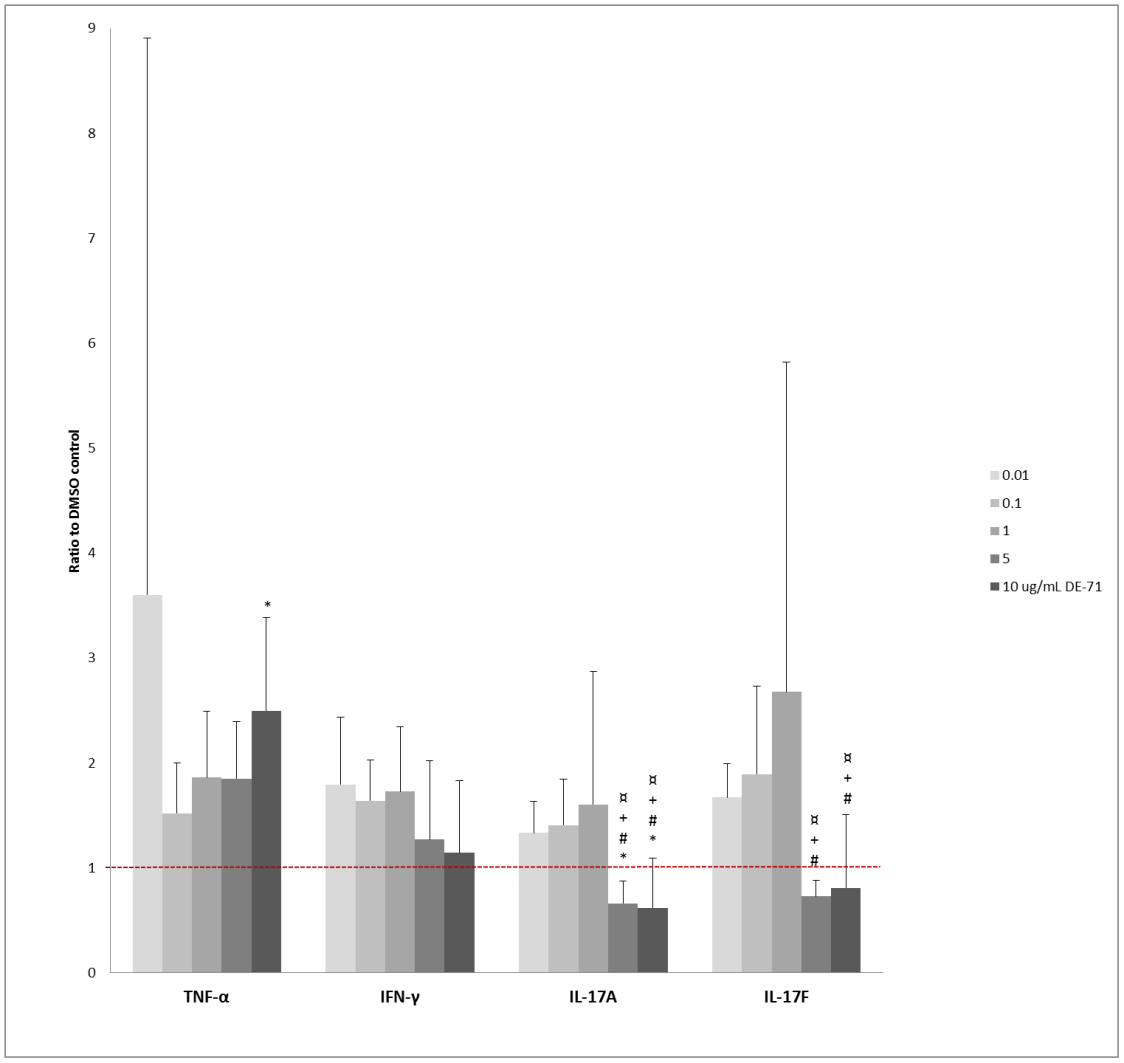
Influence of DE-71 on cytokine responses of PBMCs. Mean + SD of secretion of TNF-α, IFN-γ, IL-17A, and IL-17F from DE-71 exposed and PHA-L-stimulated PBMCs are shown as ratios to the respective DMSO controls. The red dotted line indicates the level of the DMSO controls (ratio = 1). * = p<0.05 compared to DMSO control, # = p<0.05 compared to 0.01 μg/mL DE-71, + = p<0.05 compared to 0.1 μg/mL DE-71, ¤ = p<0.05 compared to 1 μg/mL DE-71. PBMC: peripheral mononuclear cells, PHA-L: phytohemagglutinin-L, TNF-α: tumor necrosis factor-α, IFN-γ: interferon-γ, IL-17A: interleukin-17a, IL-17F: interleukin-17F DMSO = dimethyl sulfoxide, SD = standard deviation.

The cytokine concentrations (mean of duplicates) were analysed by two-way ANOVA followed by Tukey’s honest significant difference post hoc analysis. Data was Ln-transformed when necessary. Relevant results of Tukey’s post hoc analysis are shown in Figs 1 and 2, described in Results and listed in Table A and B in S1 File with p-values and 95% confidence intervals (CI). The negative controls were compared by paired t-tests. No statistical analysis was performed for cytotoxicity assessment, since only two cell experiments were done. However, cytotoxicity data are presented in graphs as means ± SD. Results were considered statistically significant when p < 0.05.

## Results

### Cytokine Secretion from Unstimulated Cells

In culture medium from unstimulated PBMCs, the levels of all cytokines (IL-1β, IL-2, IL-4, IL-6, CXCL8, IL-10, IL-17A, IL-17F, TNF-α, and INF-γ) were far below levels of LPS- or PHA-L-stimulated cells, and were not further investigated (data not shown).

### Cytokine Secretion from LPS-Stimulated PBMCs

Median and ranges for the DMSO controls with LPS stimulation are shown in Table 1. DE-71 significantly increased the secretion of IL-1β, IL-6, CXCL8, IL-10, and TNF-α (p = 0.005, p = 0.019, p = 0.012, p = 0.009, and p<0.001, respectively; n = 6) from the LPS-stimulated PBMCs, as shown in Fig 1. DMSO had no effect on cytokine secretion compared to medium alone (IL-1β: p = 0.72, IL-6: p = 0.33, IL-8: p = 0.08, IL-10: p = 0.45, TNF-α: p = 0.53) (n = 6 cultures in duplicate, data not shown).

**Table 1:** Median and range of DMSO controls from LPS-stimulated PBMCs (n = 6 cultures in duplicates).

Stimulation	Cytokine	Median (pg/mL)	Range (pg/mL)
LPS	IL-1β	198	80–1064
	IL-10	48	7.4–183
	TNF-α	887	451–2065
	CXCL8	42930	13037–84622
	IL-6	6284	1944–15582

Concentrations of INF-γ, IL-2, IL-4, IL-17A, and IL-17F were generally below the lowest standard, and therefore no further analyses of these data were performed (data not shown).

Post-hoc analysis showed a significant increase of IL-1β, IL-6, CXCL8, IL-10, and TNF-α secretion in cultures exposed to 5 and 10 μg/mL DE-71, compared to controls. A few other DE-71 concentrations also caused significant increases in TNF-α secretion (Fig 1, exact values are listed in Table 1 in S1 File). Three out of six cultures showed a tendency towards an increase in IL-1β, IL-10, and TNF-α upon addition of 0.01 μg/mL DE-71, compared to relevant controls visualised in Fig 1. Increasing the concentration further to 0.1 μg/mL caused a slight, albeit non-significant decrease in cytokine production (data not shown).

### Cytokine Secretion from PHA-L-Stimulated PBMC

Median and ranges for the DMSO controls with LPS stimulation are shown in Table 2. The differences found in the post hoc analysis are listed in Table B in S1 File. DE-71 significantly enhanced the PHA-L-elicited secretion of IFN-γ, TNF-α, IL-17A, and IL-17F (with p-values of 0.016, 0.043, < 0.001 and < 0.001, respectively; n = 6) (Fig 2), while secretion of IL-1β, IL-2, IL-10, IL-8 and IL-6 was not significantly affected by DE-71) (with p-values of 0.071, 0.19, 0.070, 0.14; n = 6 cultures in duplicates; data not shown and 0.40; n = 5 due to unmeasurable values in one experiment, respectively).

**Table 2:** Median and range of DMSO controls from PHA-L-stimulated PBMCs (n = 6 cultures in duplicates).

Stimulation	Cytokine	Median (pg/mL)	Range (pg/mL)
PHA-L	TNF-α	638	110–1414
	IFN-γ	19	8.6–534
	IL-17a	76	4.8–113
	IL-17f	493	11–1161

DMSO did not seem to affect the cytokine secretion compared to medium alone (range of p-values, 0.06–0.66), except for that of IL-17A (p = 0.024) (n = 6 cultures in duplicates, data not shown). The concentration of IL-4 was generally below the detection limit in all experiments and therefore not subjected to further analysis (data not shown).

Although the post-hoc analyses did not show any significant differences (data not shown), and a true dose-response relationship could not be defined, lower concentrations of DE-71 seemed to stimulate IFN-γ secretion, while higher concentrations were associated with IFN- γ levels similar to those of the control (Fig 2). There was a tendency towards a dose-response increase of DE-71 on TNF-α release (Fig 2). However, this was only significant for 10 μg/mL DE-71, compared to the control in the post-hoc analysis (Fig 2, Table B in S1 File). The secretion of both IL-17A and IL-17F was inhibited by 5 and 10 μg/mL DE-71, compared to the lower DE-71 concentrations, and for IL-17A also to the control (Fig 2).). Conversely, the secretion of both IL-17A and IL-17F tended to increase upon exposure of PMBCs to the lower concentrations of DE-71, but thiswas not confirmed in the post-hoc analysis.

### Cytotoxicity

**Fig 3:**
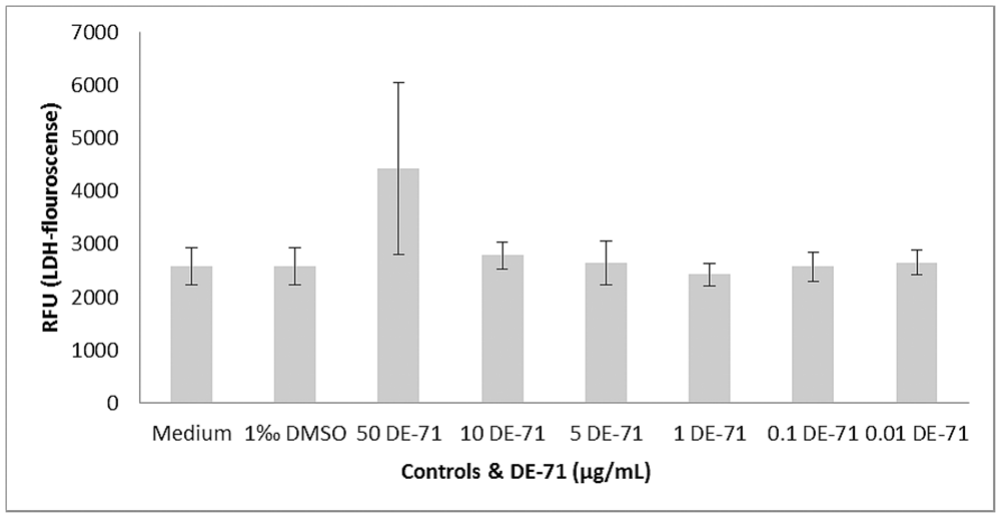
Mean of LDH-fluorescence from DE-71 exposed and LPS-stimulated PBMCs. DE-71 was added in six different concentrations to each culture and medium, and DMSO in medium served as negative controls in duplicate, n = 2 cultures in triplicates. LDH: lactate dehydrogenase, LPS: lipopolysaccharide, DMSO: dimethyl sulfoxid, RFU: relative fluorescence unit.

**Fig 4:**
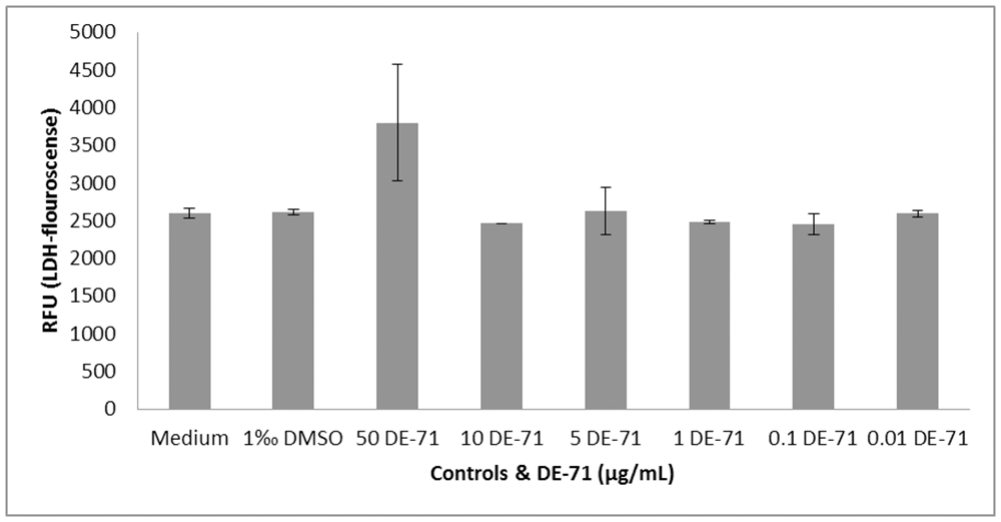
Mean of LDH-fluorescence from DE-71 exposed and PHA-L-stimulated PBMCs. DE-71 was added in six different concentrations to each culture, medium and DMSO in medium served as negative controls in duplicate, n = 2 cultures in triplicates. LDH: lactate dehydrogenase, PHA-L: phytohemagglutinin-L, DMSO: dimethyl sulfoxid, RFU: relative fluorescence unit.

DE-71, at a concentration of 50 μg/mL, caused an increase in LDH-levels compared to the negative control, while no increase was observed at lower DE-71 concentrations. DMSO had no effect on cytotoxicity compared to culture medium controls (Fig 3 + Fig 4).

### Endotoxin Test

All results were negative.

#### PBDE analysis

The PBDE analysis showed a consistent congener pattern in all samples, the dominating congener being BDE-99 (50.1%), followed by BDE-47 (28,5%) and BDE-100 (8,9%). The concentration of DE-71, calculated as the sum of the PBDE congeners, in three stock solutions and in culture medium deviated slightly from the expected concentrations (Table 3). The deviation increased throughout the serial dilution. The spiked control samples analysed along with the DMSO solutions, ranged from 102% (BDE-100) to 107% (BDE-183) (mean of four control samples).

**Table 3:** PBDE measurements in stock solution, culture media and cells, mean of double determinations.

Sample type	Measured concentration	
**Stockk sollutions (mg/mL)**	4.2	
	7.4	
	0.03	
**Culture media (μg/mL)**	2.6	
	0.2	
**Cells after exposure (μg/mL)**	**Supernatant**	**Cell remnants**
	1.7 a)	1.6
	0.008	0.003

^a)^ The concentration was outside the calibration range and thus likely underestimated.

Analysis of cell samples and supernatants showed that the majority of DE-71 was present in the supernatant. At a concentration of 0.01 μg/mL, DE-71 could be recovered completely from the chemical analysis, while the absence of a valid result for the supernatant of cultures stimulated with 10 μg/mL DE-71 made the mass balance calculation for this sample more uncertain. The spiked control samples extracted together with the samples ranged from 80% (BDE-183) to 96% (BDE-47) (mean of three control samples). The analyses of controls containing only DMSO showed trace levels of BDE-99 (< 0.2 ng/mL).

## Discussion

The effect of PBDEs on the immune system is largely unknown. In this study, we have demonstrated an enhancing, non-cytotoxic effect of DE-71 (0.01–10 μg/mL) on cytokine production by normal human PBMCs stimulated with LPS or PHA-L *ex vivo*.

The cytokine pattern found for LPS-stimulated cells indicated that DE-71 enhanced pro-inflammatory responses by monocytes and macrophages, reflected by increasing secretion of TNF-α, IL-1β, IL-6, and IL-8. Secretion of the anti-inflammatory IL-10 was increased as well, possibly reflecting a subsequent compensatory down-regulation of an anti-inflammatory response [23, 24]. Thus, DE-71 appeared to enhance pro-inflammatory responses by innate immune cells.

To examine the effect of DE-71 specifically on the adaptive immune system, PBMCs were stimulated with PHA-L. DE-71 tended to enhance PHA-L-induced IFN-γ responses at low concentrations (0.01–1 μg/mL) (Fig 2), supporting a promotion of Th1-cell responses. This conclusion was supported by an enhancing effect of DE-71 on TNF-α production, while not supported by statistical analyses of IFN-γ responses alone. High concentrations of DE-71 caused a reduction of the PHA-L-induced IL-17A and IL-17F production, indicating suppression of Th17-cell responses, in a greement with Th17 responses usually being suppressed by Th1 cytokines [25]. The apparent skewing of T-cell responses towards a Th1-response, and away from Th17 responses might indicate that defence against infection with extracellular bacteria and fungi, which normally requires Th17 responses [26, 27], may be compromised by DE-71. It can further be speculated that inappropriate activation of the Th1 cells might facilitate development of autoimmune disease [28].

It is difficult to compare these findings with other studies, since studies concerning PBDE effects on immune cells *in vitro* and *ex vivo* are few, with a wide range of different endpoints and mixed conclusions. Only few studies have investigated cytokine responses: Koike et al. [29] found increased production of IL-4 (pg/mL) and increased antigen expression in mouse immune cells (splenocytes and bone marrow cells) after addition of five different flame retardant mixtures to final concentrations of 0.1–10 μg/mL. Koike et al. used concentrations of DE-71 that were comparable to ours, but stimulated the cells with granulocyte macrophage-stimulating factor rather than PHA-L or LPS, which may explain why their findings differed from ours. Hennigar et al. [13] found reduced TNF-α and IL-6 (pg/mL) secretion in porcine alveolar macrophages after addition of seven different concentrations of DE-71 (ranging from 0.0001 μg/mL to 2 μg/mL. These findings are in disagreement with our finding of a lack of effect on IL-4 (pg/mL), and enhancement of TNF-α and IL-6 responses. We used higher concentrations of DE-71 than Hennigar et al. (who used 0.1–2000 pg/mL), which may be one explanation for the diverging results.

One study examined the effect of BDE-99, the mixture Bromkal 70-5DE which is very similar to DE-71, on several cytokines (IL-1α, IL-1β, IL-2, IL-3, IL-4, IL-5, IL-6, IL-9, IL-10, IL-12, IL-13, IL-17, IFN-γ, CXCL1, MCP-1, MIP-1β, RANTES, TNFα, and VEGF) *ex vivo*, and on replication of microorganisms in coxsackievirus-infected mice [30]. PBDEs caused a drastic reduction in circulating cytokine concentrations in non-infected mice, to a higher degree than in infected mice. The discrepancy of these findings from ours may relate to differences in cell type, cell stimuli, methods of cytokine measurements, or exposure time.

Many protocols have been used to study the effects of PBDEs on human cells *in vitro*. One study investigated immunoglobulin synthesis and proliferation of lymphocytes incubated for 72 hours with two congeners, BDE-47 (4.85*10^−4^–4.85 μg/mL) and BDE-85 (5.65*10^−4^–5.65 μg/mL), i.e. at concentration ranges lower than those used in our study, and found no significant effect of these compounds [19]. Koike et al. used 24 hours incubation with DE-71 and two other commercial mixtures to examine the effect on ICAM-1, IL-6 and IL-8 expression in human bronchial epithelial cells pre-treated with protein kinase inhibitors or nuclear receptor antagonists [31]. As in the present study, they also diluted DE-71 in DMSO (1 ‰) and culture medium to final concentrations of 0.01–10 μg/mL. Neither study found any cytotoxicity at concentrations ranging from 0.01–10 μg/mL. Koike et al. measured the cytokine content by enzyme-linked immunosorbent assay, while in the current study a cytometric bead-array system was used. They found an increased production of IL-6, and IL-8, in accordance with our demonstration of an enhancement from DE-71 on the production of these mediators by LPS-stimulated cells. Thus, a proinflammatory reaction similar to that of PBMCs occurred in bronchial epithelial cells. Finally, both data and the description above indicated a direct effect of PBDEs on cytokine production.

The composition of the DE-71 mixture used in this study was in good agreeement with the profile of DE-71 specifed by La Guardia et al. [32], and remained unchanged throughout dilutions. Consistent with the chemical analysis, which only showed unquantifiable traces of PBDEs in the control samples, no traces were observed in cell controls without addition of DE-71. Our analysis further showed a partitioning of the PBDEs between cell supernatants and remnants. For one of the samples, the detectable amounts were in agreement with the expected concentrations. However, an uncertain quantification in the second data set prevented an accurate mass balance calculation of this sample. Several factors are likely to influence the PBDE recovery, in particular sorption processes. Our findings underline that the quantification of actual contaminant concentrations in the assays is important in toxicological research.

The PBMCs in our experiment were incubated with DE-71 for 20–22 hours. In comparison, we investigated accumulation of DE-71 in human thyroid cells (*unpublished)* with an incubation time of 72 hours, where ~100% of the added DE-71 was recovered fromthe cell remnants. Hence, it is plausible that DE-71 accumulates over time in human cells. The effect of different exposure times in other such experiments is unknown, and further experiments are needed to investigate these conditions.

The chosen concentrations of DE-71 were based on previous *in vitro* studies [29, 33], which exceeded PBDE levels found in the indoor environment in Denmark [10], as well as in the blood of Danish donors [22, 34]. These studies showed correlations between PBDEs in dust and blood, suggesting that dust was a primary exposure source in humans. However, it is not possible to extrapolate *in vitro* experiments to *in vivo* consequences. This might be due to reduced effective doses of biologically test chemicals *in vitro*, evaporation *in vitro* and/or non-specific binding to extracellular matrices. Furthermore, a limited number of target sites in cells reduced the numberof toxicity pathways *in vitro* compared to pathways on a multi-organ level [35]. It is important to note that a comparison between environmental exposure sources and influences on cytokines in blood *in vitro* is not directly possible. The results, however, have contributed to the elucidation of the cellular effect mechanism of a commercial flame retardant mixture in PBMCs. Lower concentration ranges are suggested in future investigations considering our results. Consequences of the demonstrated specific cytokine response should be considered and compared to cytokine levels in normo- and pathophysiological conditions.

Of major importance for the future, new flame retardant chemicals sharing structural and physico-chemical characteristics with the PBDEs have been found in dust samples in American and European homes, including samples from Denmark [36–38]. The same adverse effects as those observed after exposure to PBDEs may be expected for these new compunds but are largely unknown. Further studies should include investigations of any immunotoxic mode of action, as well as comparisons between the PBDEs and new flame retardants.

## Conclusion

Previous studies on DE-71 have suggested that it can influence the immune system in various ways. Our results support this assumption: we showed that LPS- and PHA-L-induced cytokine secretions by innate immune cells and T cells, respectively, were influenced by DE-71, at non-cytotoxic concentrations (0.01–10 μg/mL), *in vitro*. DE-71 appeared to have an enhancing effect on innate immune responses, as the production of all of the cytokines induced by LPS stimulation were increased. Moreover, our study indicates that DE-71 promotes Th1 responses and inhibits Th17 responses. These data thus add to earlier findings on cytotoxicity and pro-inflammatory responses in human and animal cells. However, cytokine measurements have been scarce and have in previous studies only dealt with few different cytokines per study. More studies on the cytokine secretion patterns after DE-71 exposure are therefore warranted.

## Supporting Information

S1 File Table A and Table B. Post-hoc analyses of significant ANOVA-results.
(DOCX)
