# Supplementary material for: Effects of the Commercial Flame Retardant Mixture DE-71 on Cytokine Production by Human Immune Cells
Source: PLoS One. 2016 Apr 29;11(4):e0154621. doi: 10.1371/journal.pone.0154621 (PMC4851365; doi:10.1371/journal.pone.0154621)
Supplement: S1 File — Post-hoc analyses of significant ANOVA-results. (DOCX) [file pone.0154621.s001.docx]

**Supporting Information**
**Table A. Results from post-hoc analyses of significant ANOVA-results from LPS- and DE-71 stimulated cells.** LPS: lipopolysaccharide, ANOVA: analysis of variance, CI: confidence intervals.

| **TukeyHSD results** | **Transformation of data** | **Exposure Comparisons** | **Estimated ratios/differences (95% CI)** | **Tukey comparisons p-value** |
| --- | --- | --- | --- | --- |
| IL-1β | ln | 5 μg/mL vs. control | 2.7 (1.2; 5.4) | 0.011 |
|  |  | 10 μg/mL vs. control | 2.9 (1.3;6.6) | 0.004 |
| IL-10 | ln | 5 μg/mL vs. control | 2.6 (1.2; 5.6) | 0.007 |
|  |  | 10 μg/mL vs. control | 2.5 (1.2; 5.2) | 0.13 |
| TNF-α | ln | 1 μg/mL vs. control | 1.4 (1.0; 2.0) | 0.035 |
|  |  | 5 μg/mL vs. control | 1.8 (1.3; 2.6) | 0.0001 |
|  |  | 10 μg/mL vs. control | 1.8 (1.3; 2.5) | 0.00017 |
|  |  | 5 μg/mL vs. 0.1 μg/mL | 1.5 (1.1; 2.1) | 0.019 |
|  |  | 10 μg/mL vs. 0.1 μg/mL | 1.5 (1.0; 2.0) | 0.023 |
| IL-8 | ln | 5 μg/mL vs. control | 1.9 (1.1; 3.5) | 0.024 |
|  |  | 10 μg/mL vs. control | 1.8 (1.0; 3.3) | 0.045 |
| IL-6 | none | 5 μg/mL vs. control | 4970 (559;9381) | 0.021 |
|  |  | 10 μg/mL vs. control | 5031 (620;9442) | 0.019 |

**Table B. Results from Post-hoc analyses of significant ANOVA-results from PHA-L- and DE-71 stimulated cells.** IFN-γ did not show any significance in the Post-hoc-analysis (data not shown). PHA-L: phytohemagglutinin-L, ANOVA: analysis of variance, CI: confidence intervals.

| **TukeyHSD results** | **Transformation of data** | **Exposure Comparisons** | **Estimated ratios/differences (95% CI)** | **Tukey comparisons p-value** |
| --- | --- | --- | --- | --- |
| TNF-α | none | 10 μg/mL vs. control | 810 (119; 1501) | 0.015 |
| IL-17A | none | 5 μg/mL vs. control | -29 (-59; -1) | 0.041 |
|  |  | 10 μg/mL vs. control | -37 (-65; -8) | 0.007 |
|  |  | 5 μg/mL vs. 0.01 μg/mL | -51 (-79; -22) | 0.0002 |
|  |  | 10 μg/mL vs. 0.01 μg/mL | -58 (-86; -29) | <0.0001 |
|  |  | 5 μg/mL vs. 0.1 μg/mL | -48 (-76; -19) | 0.0003 |
|  |  | 10 μg/mL vs. 0.1 μg/mL | -55 (-83; -26) | 0.0001 |
|  |  | 5 μg/mL vs. 1 μg/mL | -38 (-67; -9) | 0.005 |
|  |  | 10 μg/mL vs. 1 μg/mL | -45 (-74; -16) | 0.0007 |
| IL-17F | ln | 5 μg/mL vs. 0.01 μg/mL | 0.44 (0.21; 0.89) | 0.017 |
|  |  | 10 μg/mL vs. 0.01 μg/mL | 0.37 (0.18; 0.75) | 0.003 |
|  |  | 5 μg/mL vs. 0.1 μg/mL | 0.40 (0.20; 0.83) | 0.008 |
|  |  | 10 μg/mL vs. 0.1 μg/mL | 0.34 (0.16; 0.69) | 0.001 |
|  |  | 5 μg/mL vs. 1 μg/mL | 0.39 (0.19; 0.80) | 0.005 |
|  |  | 10 μg/mL vs. 1 μg/mL | 0.33 (0.16; 0.67) | 0.001 |
